# NNLO interpolation grids for jet production at the LHC

**DOI:** 10.1140/epjc/s10052-022-10880-2

**Published:** 2022-10-19

**Authors:** D. Britzger, A. Gehrmann-De Ridder, T. Gehrmann, E. W. N. Glover, C. Gwenlan, A. Huss, J. Pires, K. Rabbertz, D. Savoiu, M. R. Sutton, J. Stark

**Affiliations:** 1 Max-Planck-Institut für Physik, Föhringer Ring 6, 80805 Munich, Germany; 2 Institute for Theoretical Physics, ETH, Wolfgang-Pauli-Strasse 27, 8093 Zurich, Switzerland; 3 Physik-Institut, Universität Zürich, Winterthurerstrasse 190, 8057 Zurich, Switzerland; 4 Institute for Particle Physics Phenomenology, Durham University, Durham, DH1 3LE UK; 5 Department of Physics, The University of Oxford, Oxford, OX1 3RH UK; 6 Theoretical Physics Department, CERN, 1211 Geneva 23, Switzerland; 7 LIP, Avenida Professor Gama Pinto 2, 1649-003 Lisbon, Portugal; 8 Faculdade de Ciências, Universidade de Lisboa, 1749-016 Lisbon, Portugal; 9 Institut für Experimentelle Teilchenphysik (ETP), KIT, Wolfgang-Gaede-Str. 1, 76131 Karlsruhe, Germany; 10 Experimental Physics Department, CERN, 1211 Geneva 23, Switzerland; 11 Institut für Experimentalphysik, Universität Hamburg, Luruper Chaussee 149, 22761 Hamburg, Germany; 12 Department of Physics and Astronomy, The University of Sussex, Brighton, BN1 9RH UK

## Abstract

Fast interpolation-grid frameworks facilitate an efficient and flexible evaluation of higher-order predictions for any choice of parton distribution functions or value of the strong coupling $\alpha _{\mathrm {s}}$. They constitute an essential tool for the extraction of parton distribution functions and Standard Model parameters, as well as studies of the dependence of cross sections on the renormalisation and factorisation scales. The APPLfast project provides a generic interface between the parton-level Monte Carlo generator  and both the APPLgrid and the fastNLO libraries for the grid interpolation. The extension of the project to include hadron–hadron collider processes at next-to-next-to-leading order in perturbative QCD is presented, together with an application for jet production at the LHC.

## Introduction

Theory predictions at next-to-next-to-leading order (NNLO) in perturbative QCD (pQCD) are the current new standard for an increasingly large range of important LHC processes [1]. This development is underscored by the completion of almost all relevant $2\rightarrow 2$ scattering processes at this order and first results for genuine $2\rightarrow 3$ processes, see e.g. Ref. [2] for a recent overview. The  program [3] provides a single framework for performing such calculations fully differentially, and is under continuous development to provide state-of-the-art theory predictions for a plethora of processes.

Despite this remarkable progress, applications of these calculations, beyond simple predictions, still remain the exception: computational efficiency is the primary bottleneck that restricts the wide-spread use of NNLO calculations in applications such as the extraction of parton distribution functions (PDFs) [4] or Standard Model (SM) parameters such as $\alpha _{\mathrm {s}}$  [5] or a thorough assessment of theoretical uncertainties. With a typical computing cost that exceeds $\mathcal {O}(10^5)$ CPU core hours, any application that relies on the repeated calculation of the cross section with different input conditions—e.g. the variation of the value of the strong coupling $\alpha _{\mathrm {s}}$, the parametrisation of PDFs, or the study of the dependence on the renormalisation and factorisation scales—quickly becomes a formidable challenge.

The technique of fast interpolation grids [6] addresses this bottleneck and has been implemented in the APPLgrid  [7, 8] and fastNLO  [9, 10] packages. The extension of this approach to NNLO predictions has been achieved for the case of DIS in Ref. [11, 12] and diffractive DIS in Ref. [13]. First applications of the grid technique to hadron–hadron collision processes at NNLO were discussed in Refs. [14–18]. In this paper, the approach is extended to include the NNLO processes in hadron–hadron collisions available from the  program. The technique has been applied to the important and complex process of jet production at LHC energies, and is here described in detail. The application to jet production cross sections can be seen as a proof-of-principle for any further processes implemented in  at NNLO QCD.

The paper is structured as follows: Sect. 2 provides a brief review of the grid technique, whilst highlighting the main conceptual differences with respect to the DIS case and providing additional necessary details of the implementation; the interpolation quality is discussed in Sect. 3 and the inclusive jet production process is used as an example. Various sources of theoretical uncertainties are considered, including those due to the scale, $\alpha _{\mathrm {s}}$, and the PDF, followed by an assessment of the quality of the commonly employed $K$-factor approach, and an investigation of the total fiducial inclusive jet cross section at the LHC. In Sect. 4, a simultaneous $\alpha _{\mathrm {s}}$ and PDF fit is performed using the dijet process. Grids are made publicly available on the Ploughshare website [19].

## NNLO predictions for hadron–hadron colliders and the APPLfast project

### Differential predictions

Cross section predictions for hadron–hadron collisions are described through QCD factorisation as a convolution of the underlying hard scattering of partons and the PDFs for each target hadron,

1 $$\begin{aligned} \sigma&=\int \mathrm {d}x_1 \mathrm {d}x_2 f_a(x_1,\mu _\mathrm{F}) f_b(x_2,\mu _\mathrm{F})\nonumber \\&\quad \times \mathrm {d}\hat{\sigma }_{ab}(x_1,x_2,\mu _\mathrm{R},\mu _\mathrm{F})\, , \end{aligned}$$

where an implicit summation over the incoming parton flavours *a* and *b* is assumed. The hard-scattering cross section can be obtained in pQCD as an expansion in the strong coupling

$$\begin{aligned}&\mathrm {d}\hat{\sigma }_{ab}(x_1,x_2,\mu _\mathrm{R},\mu _\mathrm{F}) \\&\qquad =\sum _{n=0}^{n_\mathrm {max}} \left( \frac{\alpha _{\mathrm {s}} (\mu _\mathrm{R})}{2\pi }\right) ^{p+n} \mathrm {d}\hat{\sigma }^{(n)}_{ab}(x_1,x_2,\mu _\mathrm{R},\mu _\mathrm{F}) \, , \end{aligned}$$

where *p* denotes the power in $\alpha _{\mathrm {s}}$ of the leading-order (LO) process ($n=0$) and $n_\mathrm {max}$ the number of orders beyond LO that are considered in the perturbative calculation.

Predictions beyond Born level ($n>0$) receive contributions that involve additional loop integrations and real-emission corrections. This gives rise to a set of parton-level ingredients of different particle multiplicities that are individually divergent and only finite in their sum for sufficiently inclusive quantities. Fully differential predictions must retain the full kinematic information of the final state while at the same time ensuring the cancellation of such infrared singularities. The calculations within the  framework [3] accomplish this task through the re-distribution of singularities using the antenna subtraction formalism [20–22]. Arbitrary collinear- and infrared-safe observables can be computed in this framework through a flexible parton-level Monte Carlo generator that samples the available phase space $(x_{1;m},x_{2;m},\varPhi _m)_{m=1,\ldots ,M_n}$ with $M_n$ points and accumulates the associated weights $w^{(n)}_{ab;m}$. The cross section in Eq. (1) can then be computed via

2 $$\begin{aligned} \sigma&\xrightarrow {\text {MC}} \sum _{n=0}^{n_\mathrm {max}} \sum _{m=1}^{M_n} \left( \frac{\alpha _{\mathrm {s}} (\mu _\mathrm{R} {}_{;m})}{2\pi }\right) ^{p+n} \nonumber \\&\qquad \times f_{a}(x_{1;m}, \mu _\mathrm{F} {}_{;m}) \; f_{b}(x_{2;m}, \mu _\mathrm{F} {}_{;m}) \nonumber \\&\qquad \times w^{(n)}_{ab;m} \; \mathrm {d}\hat{\sigma }^{(n)}_{ab;m} \, , \end{aligned}$$

using the short-hand notation

$$\begin{aligned} \mu _{X;m}&\equiv \mu _X(\varPhi _m) \quad \text {for }X=\mathrm {R},\,\mathrm {F},\\ \mathrm {d}\hat{\sigma }^{(n)}_{ab;m}&\equiv \mathrm {d}\hat{\sigma }^{(n)}_{ab}(x_{1;m}, x_{2;m}, \mu _\mathrm{R} {}_{;m},\mu _\mathrm{F} {}_{;m}) \,. \end{aligned}$$

further provides a decomposition of the logarithmic structure of the cross section

3 $$\begin{aligned}&\mathrm {d}\hat{\sigma }^{(n)}_{ab} (\mu _\mathrm{R} ^2,\mu _\mathrm{F} ^2) = \sum _{{\begin{array}{c} \alpha , \beta \\ \alpha +\beta \le n \end{array}}} \mathrm {d}\hat{\sigma }^{(n | \alpha ,\beta )}_{ab} \ln ^{\alpha }\left( \frac{\mu _\mathrm{R} ^2}{\mu _0^2}\right) \ln ^{\beta } \left( \frac{\mu _\mathrm{F} ^2}{\mu _0^2}\right) \, , \end{aligned}$$

to facilitate a flexible reconstruction of the scale dependence. Here, $\mu _0$ denotes an arbitrary hard reference scale used for this decomposition.

### The grid technique for hadron–hadron collisions

The grid technique for hadron–hadron collisions at NNLO is a non-trivial extension of that for DIS [12] taking into account an additional parton distribution for the second target hadron and the corresponding momentum fraction, $x_2$. Adopting the same notation as in Ref. [12], the equivalent of Eq. (11) from there for the hadron–hadron case becomes

4 $$\begin{aligned}&\alpha _{\mathrm {s}} (\mu ) \; f_a(x_1,\mu ) f_b(x_2,\mu ) \nonumber \\&\quad \simeq \sum _{i,j,k} \alpha _{\mathrm {s}} ^{[k]} \; f^{[i,k]}_{a} f^{[j,k]}_{b} \; E^y_i(x_1) E^y_j(x_2) \; E^\tau _k(\mu ) \, , \end{aligned}$$

where $\mu _\mathrm{R} =\mu _\mathrm{F} \equiv \mu$ has been set for simplicity. The summation over *i*, *j*, and *k* represents the summation over the nodes of the grid structure for $x_1$, $x_2$, and $\mu$, respectively, where one dimension in the grid is needed for each interpolated parameter. The superscripts on the interpolation kernels $E_i^y(x)$ denote variable transformations $x \longmapsto y(x)$ that are introduced to allow for a more optimal span of the phase space of the transformed variable and to improve the interpolation quality with respect to using equidistant grid nodes. Some common choices for the transformations are given explicitly in Ref. [12].

The naïve application of the sum over the parton flavours in Eq. (4) results in up to 121 ($11\times 11$) different parton–parton luminosity contributions, or 169 if the top quark is also included, which makes the representation as a numerical grid excessively large and potentially prohibitive for practical applications. It is therefore expedient to instead make use of symmetries within the structure of the hard subprocesses to form linear combinations of the individual parton–parton luminosities to arrange for a smaller set of unique luminosities. This allows the summation over the full set of parton flavour combinations (*a* and *b*) to be replaced by a single summation over a significantly smaller set of contributions, $F_\lambda (x_1,x_2,\mu )$, such that

5 $$\begin{aligned}&\sum _\lambda F_\lambda (x_1,x_2,\mu ) \; h_\lambda (x_1,x_2, \mu ) \nonumber \\&\quad \equiv \sum _{a,b} f_a(x_1,\mu ) f_b(x_2,\mu ) \; h_{ab}(x_1,x_2, \mu ), \end{aligned}$$

where the summations over parton luminosities have been included explicitly on this occasion. The weights (denoted as *h*) for any specific contribution $\lambda$ are identical for each of the individual terms (*a*, *b*) in the summation for the corresponding index $\lambda$. As an example, the decomposition within  for jet production in hadron–hadron collisions [23–26] is

6 $$\begin{aligned} \begin{aligned} F_{1}&= f_{g}(x_1) \, f_{g}(x_2) , \\ F_{2}&= \sum _{i=1}^{5} f_{q_i}(x_1) \, f_{g}(x_2) ,&F_{\bar{2}}&= \sum _{i=1}^{5} f_{\bar{q}_i}(x_1) \, f_{g}(x_2) \\* F_{3}&= \sum _{i=1}^{5} f_{g}(x_1) \, f_{q_i}(x_2) ,&F_{\bar{3}}&= \sum _{i=1}^{5} f_{g}(x_1) \, f_{\bar{q}_i}(x_2) \\ F_{4}&= \sum _{i=1}^{5} f_{q_i}(x_1) \, f_{\bar{q}_i}(x_2) ,&F_{\bar{4}}&= \sum _{i=1}^{5} f_{\bar{q}_i}(x_1) \, f_{q_i}(x_2) \\* F_{5}&= \sum _{i=1}^{5} f_{q_i}(x_1) \, f_{q_i}(x_2) ,&F_{\bar{5}}&= \sum _{i=1}^{5} f_{\bar{q}_i}(x_1) \, f_{\bar{q}_i}(x_2) \\ F_{6}&= \sum _{i,j=1}^{5} f_{q_i}(x_1) \, f_{\bar{q}_j}(x_2) ,&F_{\bar{6}}&= \sum _{i,j=1}^{5} f_{\bar{q}_i}(x_1) \, f_{q_j}(x_2) \\* F_{7}&= \sum _{i,j=1}^{5} f_{q_i}(x_1) \, f_{q_j}(x_2) ,&F_{\bar{7}}&= \sum _{i,j=1}^{5} f_{\bar{q}_i}(x_1) \, f_{\bar{q}_j}(x_2) , \end{aligned} \end{aligned}$$

effectively reducing the number of separate contributions that must be stored in the grid from up to 121 down to 13. This reduction of the parton luminosities is automatically performed in APPLfast for any hadron–hadron process based on the process-dependent implementation in . For jet production this number could in principle be further reduced down to 7 independent combinations [8].

Using this reduced number of parton luminosities, the interpolated cross section prediction can be written as

7 $$\begin{aligned} \sigma&\simeq \sum _{n} \sum _{i,j,k} \biggl (\frac{\alpha _{\mathrm {s}} ^{[k]}}{2\pi }\biggr )^{p+n} F^{[i,j,k]}_{\lambda } \; \hat{\sigma }^{(n)}_{\lambda [i,j,k]} \, , \end{aligned}$$

where the summation over $\lambda$ is implied. The corresponding grid is obtained by accumulating the weights according to

8 $$\begin{aligned}&\hat{\sigma }^{(n)}_{\lambda [i,j,k]} \xrightarrow {\text {MC}} \nonumber \\&\qquad \sum _{m=1}^{M_n} E^y_i(x_{1;m}) E^y_j(x_{2;m}) E^\tau _k(\mu _{m}) \; w^{(n)}_{\lambda ;m} \; \mathrm {d}\hat{\sigma }^{(n)}_{\lambda ;m} \, , \end{aligned}$$

where now the terms $w^{(n)}_{\lambda ;m}$ correspond to those weights $w^{(n)}_{ab;m}$ associated with the individual terms for $\lambda$.

### Renormalisation and factorisation scale dependence

A flexible variation of the renormalisation and factorisation scales in NNLO pQCD predictions is important for many phenomenological applications. The interpolation grids developed here allow the variation of the scales by arbitrary factors, and a selection of different scale choices, without a recalculation of the hard coefficients. With the hard coefficients $\hat{\sigma }^{(n)}_{\lambda [i,j,k]}$ determined separately order by order in $\alpha _{\mathrm {s}}$, the dependence on the renormalisation and factorisation scales, $\mu _\mathrm{R}$ and $\mu _\mathrm{F}$, can be restored using the RGE running of $\alpha _{\mathrm {s}}$ and the DGLAP evolution for the PDFs. Introducing a generic functional form depending on the scale choice $\mu$ during the grid generation in Eq. (8),

9 $$\begin{aligned} \mu _X&= \mu _X(\mu ) \quad \text {for }X=\mathrm {R},\,\mathrm {F} \,, \end{aligned}$$

and using the short-hand notation from Ref. [12],

$$\begin{aligned} L^{[k]}_{\mathrm {X}}&\equiv \ln \left( \frac{\mu _X^2(\mu ^{[k]})}{\mu ^{2[k]}}\right) \quad \text {for }X=\mathrm {R},\,\mathrm {F},\\ \alpha _{\mathrm {s}} ^{[k_{\rightarrow \mathrm {R}}]}&\equiv \alpha _{\mathrm {s}} (\mu _\mathrm{R} (\mu ^{[k]})) \, , \quad \text {and} \\ F^{[i,j,k_{\rightarrow \mathrm {F}}]}_{\lambda }&\equiv F_\lambda (x_1^{[i]}, x_2^{[j]},\mu _\mathrm{F} (\mu ^{[k]})) \, , \end{aligned}$$

the full scale dependence up to NNLO is then given by [23]

10 $$\begin{aligned}&\sigma ^\text {NNLO}(\mu _\mathrm{R},\mu _\mathrm{F})\nonumber = \sum _{i,j,k} \biggl (\frac{\alpha _{\mathrm {s}} ^{[k_{\rightarrow \mathrm {R}}]}}{2\pi }\biggr )^{p} F^{[i,j,k_{\rightarrow \mathrm {F}}]}_\lambda \; \hat{\sigma }^{(0)}_{\lambda [i,j,k]} \nonumber \\&\quad +\sum _{i,j,k} \biggl (\frac{\alpha _{\mathrm {s}} ^{[k_{\rightarrow \mathrm {R}}]}}{2\pi }\biggr )^{p+1} \biggl \{ F^{[i,j,k_{\rightarrow \mathrm {F}}]}_\lambda \; \hat{\sigma }^{(1)}_{\lambda [i,j,k]} \nonumber \\&\qquad + \Bigl [ p \beta _0 F^{[i,j,k_{\rightarrow \mathrm {F}}]}_\lambda L^{[k]}_{\mathrm {R}} \nonumber \\&\qquad \quad -\left( F_{\lambda ;{f_a\rightarrow P_0\otimes f_a}}^{[i,j,k_{\rightarrow \mathrm {F}}]} + F_{\lambda ;{f_b\rightarrow P_0\otimes f_b}}^{[i,j,k_{\rightarrow \mathrm {F}}]} \right) L^{[k]}_{\mathrm {F}} \Bigr ] \; \hat{\sigma }^{(0)}_{\lambda [i,j,k]} \biggr \} \nonumber \\ {}&\quad +\sum _{i,j,k} \biggl (\frac{\alpha _{\mathrm {s}} ^{[k_{\rightarrow \mathrm {R}}]}}{2\pi }\biggr )^{p+2} \biggl \{ F_\lambda ^{[i,j,k_{\rightarrow \mathrm {F}}]} \; \hat{\sigma }^{(2)}_{\lambda [i,j,k]} \nonumber \\ {}&\qquad + \Bigl [ (p+1) \beta _0 F^{[i,j,k_{\rightarrow \mathrm {F}}]}_\lambda L^{[k]}_{\mathrm {R}} \nonumber \\&\qquad \quad \;-\left( F_{\lambda ;{f_a\rightarrow P_0\otimes f_a}}^{[i,j,k_{\rightarrow \mathrm {F}}]} +F_{\lambda ;{f_b\rightarrow P_0\otimes f_b}}^{[i,j,k_{\rightarrow \mathrm {F}}]} \right) L^{[l]}_{\mathrm {F}} \Bigr ] \; \hat{\sigma }^{(1)}_{\lambda [i,j,k]} \nonumber \\&\qquad + \Bigl [ \Bigl ( p \beta _1 + \tfrac{1}{2}p(p+1)\beta _0^2 L^{[k]}_{\mathrm {R}} \Bigr ) \; F^{[i,j,k_{\rightarrow \mathrm {F}}]}_\lambda L^{[j]}_{\mathrm {R}} \nonumber \\&\qquad \quad \;- \left( F_{\lambda ;{f_a\rightarrow P_1\otimes f_a}}^{[i,j,k_{\rightarrow \mathrm {F}}]} +F_{\lambda ;{f_b\rightarrow P_1\otimes f_b}}^{[i,j,k_{\rightarrow \mathrm {F}}]} \right) L^{[k]}_{\mathrm {F}} \nonumber \\&\qquad \quad \;+\tfrac{1}{2} \left( F_{\lambda ;{f_a\rightarrow P_0\otimes P_0\otimes f_a}}^{[i,j,k_{\rightarrow \mathrm {F}}]} + F_{\lambda ;{f_b\rightarrow P_0\otimes P_0\otimes f_b}}^{[i,j,k_{\rightarrow \mathrm {F}}]} \right) L^{2[k]}_{\mathrm {F}} \nonumber \\&\qquad \quad \,+ \Bigl (\tfrac{1}{2} \beta _0 L^{[j]}_{\mathrm {F}} -(p+1) \beta _0 L^{[k]}_{\mathrm {R}} \Bigr )\nonumber \\&\qquad \quad \quad \;\times \left( F_{\lambda ;{f_a\rightarrow P_0\otimes f_a}}^{[i,j,k_{\rightarrow \mathrm {F}}]} +F_{\lambda ;{f_b\rightarrow P_0\otimes f_b}}^{[i,j,k_{\rightarrow \mathrm {F}}]} \right) L^{[k]}_{\mathrm {F}} \nonumber \\&\qquad \quad \;+ F_{\lambda ;{f_a\rightarrow P_0\otimes f_a};{f_b\rightarrow P_0\otimes f_b}}^{[i,j,k_{\rightarrow \mathrm {F}}]} L^{2[k]}_{\mathrm {F}} \Bigr ] \; \hat{\sigma }^{(0)}_{\lambda [i,j,k]} \biggr \}\, . \end{aligned}$$

Here, the notation $F_{\lambda ;{f_a\rightarrow X_a}}$ represents the term $F_\lambda$ but with $f_a$ replaced by $X_a$. In APPLgrid, the calculation of the scale-dependent terms is performed only if and when required, with the convolutions involving the splitting functions $P_{n}$ evaluated using Hoppet  [27].

As an alternative to the analytical reconstruction of the scale variation in Eq. (10), additional individual grids for each scale-independent coefficient can be generated, which then are multiplied with the scale-dependent logarithms. This corresponds to the default strategy in the fastNLO library where the full scale dependence is reconstructed using

11 $$\begin{aligned}&\sigma ^\text {NNLO}(\mu _\mathrm{R},\mu _\mathrm{F}) = \sum _{i,j,k} \biggl (\frac{\alpha _{\mathrm {s}} ^{[k_{\rightarrow \mathrm {R}}]}}{2\pi }\biggr )^{p} F^{[i,j,k_{\rightarrow \mathrm {F}}]}_{\lambda } \; \hat{\sigma }^{(0|0,0)}_{\lambda [i,j,k]} \nonumber \\&\quad +\sum _{i,j,k} \biggl (\frac{\alpha _{\mathrm {s}} ^{[k_{\rightarrow \mathrm {R}}]}}{2\pi }\biggr )^{p+1} F^{[i,j,k_{\rightarrow \mathrm {F}}]}_{\lambda } \; \nonumber \\&\qquad \times \biggl \{ \hat{\sigma }^{(1|0,0)}_{\lambda [i,j,k]} + L^{[k]}_{\mathrm {R}} \; \hat{\sigma }^{(1|1,0)}_{\lambda [i,j,k]} + L^{[k]}_{\mathrm {F}} \; \hat{\sigma }^{(1|0,1)}_{\lambda [i,j,k]} \biggr \} \nonumber \\&\quad +\sum _{i,j,k} \biggl (\frac{\alpha _{\mathrm {s}} ^{[k_{\rightarrow \mathrm {R}}]}}{2\pi }\biggr )^{p+2} F^{[i,j,k_{\rightarrow \mathrm {F}}]}_{\lambda } \; \nonumber \\&\qquad \times \biggl \{ \hat{\sigma }^{(2|0,0)}_{\lambda [i,j,k]} + L^{[k]}_{\mathrm {R}} \; \hat{\sigma }^{(2|1,0)}_{\lambda [i,j,k]} + L^{[k]}_{\mathrm {F}} \; \hat{\sigma }^{(2|0,1)}_{\lambda [i,j,k]} \nonumber \\&\qquad + L^{2[k]}_{\mathrm {R}} \; \hat{\sigma }^{(2|2,0)}_{\lambda [i,j,k]} + L^{2[k]}_{\mathrm {F}} \; \hat{\sigma }^{(2|0,2)}_{\lambda [i,j,k]} \nonumber \\&\qquad + L^{[k]}_{\mathrm {R}} \; L^{[k]}_{\mathrm {F}} \; \hat{\sigma }^{(2|1,1)}_{\lambda [i,j,k]} \biggr \} \, , \end{aligned}$$

where the grids are produced in analogy to Eq. (8) but using the decomposition of Eq. (3)

$$\begin{aligned}&\hat{\sigma }^{(n|\alpha ,\beta )}_{\lambda [i,j]} \xrightarrow {\text {MC}}\\&\qquad \sum _{m=1}^{M_n} E^y_i(x_{1;m}) E^y_j(x_{2;m}) E^\tau _k(\mu _{m}) \; w^{(n)}_{\lambda ;m} \; \mathrm {d}\hat{\sigma }^{(n|\alpha ,\beta )}_{\lambda ;m} \, . \end{aligned}$$

## Applications of interpolation grids for inclusive jet production

This section presents an example of the analyses that can be performed using interpolation grids for inclusive jet production cross sections at the LHC. Measurements at 7 $\mathrm {TeV}$ by ATLAS [28] are used; the quality of the jet interpolation grids is demonstrated in Sect. 3.2, and in Sect. 3.3 the grids are used to perform a detailed comparison of scale, $\alpha _{\mathrm {s}}$, and PDF uncertainties. Section 3.4 studies the robustness of the NNLO $K$-factor approach that is commonly used in PDF fits as a proxy for the exact NNLO prediction. The grids are subsequently employed for a detailed investigation of the total inclusive jet cross section, where NNLO predictions are compared to measurements from both ATLAS and CMS.

### Interpolation grids for inclusive jet production

Inclusive jet production cross sections for the anti-$k_\text {T}$  [29] jet algorithm have been measured in proton–proton collisions by the ATLAS and CMS collaborations at different centre-of-mass energies and for different values of the jet-radius (*R*) parameter. Interpolation grids at NNLO have been generated for a large selection of these measurements and a summary of the NNLO predictions, together with their respective kinematic ranges, is provided in Table 1.^1^

**Table 1:** An overview of inclusive jet $p_\mathrm {T}$ datasets with APPLfast interpolation grids for proton–proton collisions at the LHC. For each dataset the centre-of-mass energy $\sqrt{s}$, the integrated luminosity $\mathcal {L}$, the number of data points, and the jet algorithm are listed. Jets are required to be within a given range of rapidity *y* in the laboratory frame. Available choices for the central scales for $\mu _\mathrm{R/F}$ for the interpolation grids are listed

Data	$\sqrt{s}$ [TeV]	$\mathcal {L} [\mathrm{fb}^{-1}]$	No. of points	Anti-$k_\text {T}$ *R*	Kinematic range [GeV]	Fiducial cuts	$\mu _\mathrm{R/F}$-choice
CMS [30]	2.76	0.00543	81	0.7	$p^\mathrm {jet}_\mathrm {T} \in [74,592]$	$|y|<3.0$	$p^\mathrm {jet}_\mathrm {T}$,$\hat{H}_\mathrm {T}$
ATLAS [28]	7.0	4.5	140	0.6	$p^\mathrm {jet}_\mathrm {T} \in [100,1992]$	$|y|<3.0$	$p^\mathrm {jet}_\mathrm {T}$,$\hat{H}_\mathrm {T}$
CMS [31]	7.0	5.0	133	0.7	$p^\mathrm {jet}_\mathrm {T} \in [114,2116]$	$|y|<3.0$	$p^\mathrm {jet}_\mathrm {T}$,$\hat{H}_\mathrm {T}$
ATLAS [32]	8.0	20.3	171	0.6	$p^\mathrm {jet}_\mathrm {T} \in [70,2500]$	$|y|<3.0$	$p^\mathrm {jet}_\mathrm {T}$,$\hat{H}_\mathrm {T}$
CMS [33]	8.0	5.6	248	0.7	$p^\mathrm {jet}_\mathrm {T} \in [21,74]$	$|y|<4.7$	$p^\mathrm {jet}_\mathrm {T}$,$\hat{H}_\mathrm {T}$
		19.7			$p^\mathrm {jet}_\mathrm {T} \in [74,2500]$		
ATLAS [34]	13.0	3.2	177	0.4	$p^\mathrm {jet}_\mathrm {T} \in [100,3937]$	$|y|<3.0$	$p^\mathrm {jet}_\mathrm {T}$,$\hat{H}_\mathrm {T}$
CMS [35]	13.0	36.3	$2\times 78$	0.4	$p^\mathrm {jet}_\mathrm {T} \in [97,3103]$	$|y|<2.0$	$p^\mathrm {jet}_\mathrm {T}$,$\hat{H}_\mathrm {T}$
		33.5		0.7			

For each calculation, a dedicated optimisation was employed using kinematic reweighting factors and adaptation of the phase space integration. In order to achieve a sufficient numerical accuracy, the typical statistical precision of the Monte Carlo integration is smaller than 1% in most bins, with exceptions only for some bins near the edges of the selected phase space. On modern batch computing systems, these calculations typically require between $3\cdot 10^5$ and $6\cdot 10^5$ CPU hours. Together with the generation of the interpolation grids, the usual cross sections from  are still calculated and can be used as a reference to allow numerical closure tests. The calculations are performed using either the NNPDF3.1 [36] or the CT14 [37] PDF sets at NNLO. Central scale choices of $p^\mathrm {jet}_\mathrm {T}$ and $\hat{H}_\mathrm {T}$ are available, encoded in a single interpolation grid in the case of fastNLO, or available in separate grids in the case of APPLgrid.

### Closure test

As an important first step in the use of the interpolation grids, the degree of consistency between the NNLO predictions obtained from the grids and the raw  prediction is studied. In this case, the steps in the APPLfast procedure to generate grids remain identical with the DIS case, described in Section 4 of Ref. [12]. During the execution of the calculation, in addition to the interpolation grids themselves, the usual reference predictions from  are produced, which correspond to a computation solely based on  without grid generation. For the consistency comparison of the cross section from the grid convolution with the reference cross section, the fast convolution uses the same scale and PDF choice as for the original calculation. This helps to ensure, for instance, that the density of interpolation nodes is sufficient, such that interpolation errors are negligible in comparison to the statistical uncertainty of the NNLO prediction.

**Fig. 1:**
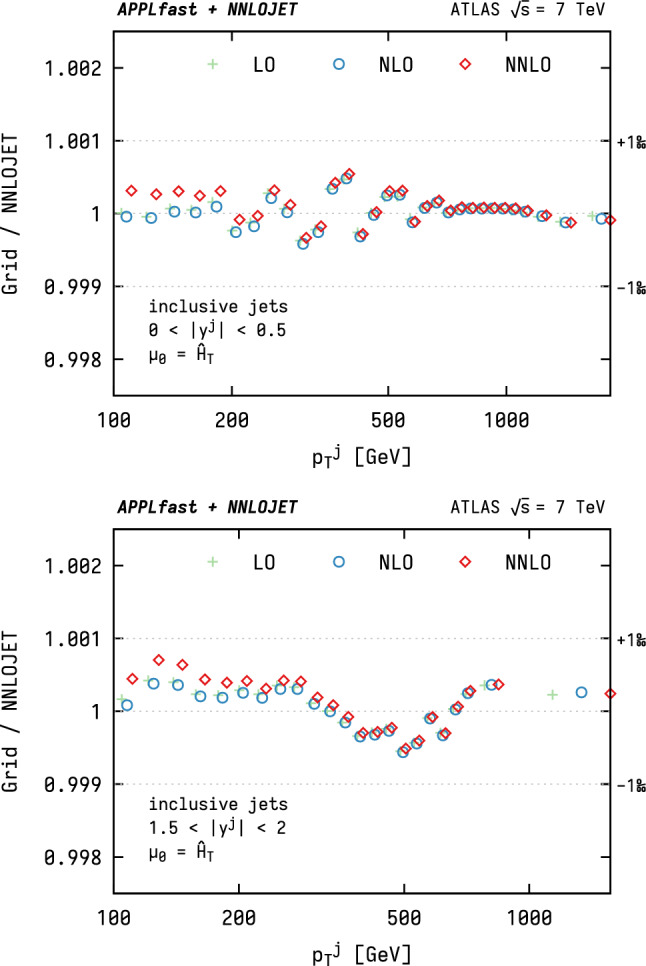
Closure tests for the ATLAS 7 TeV inclusive jet cross section grid as a function of $p^\mathrm {jet}_\mathrm {T}$ for two representative ranges in rapidity. The horizontal dotted lines indicate the targeted closure of one permille

**Fig. 2:**
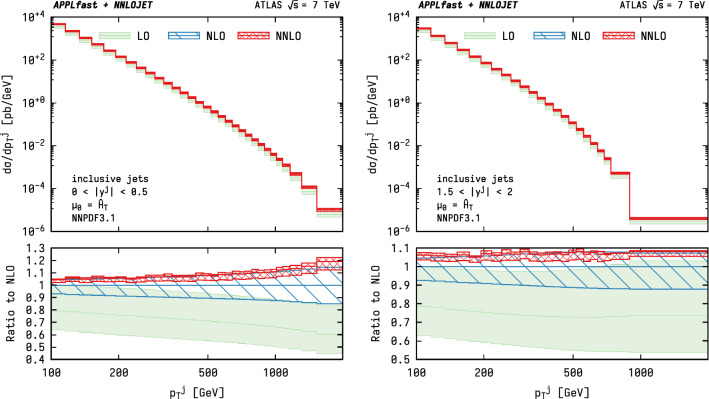
Predictions at LO, NLO and NNLO for the ATLAS 7 TeV inclusive jet cross section as a function of $p^\mathrm {jet}_\mathrm {T}$ for two representative ranges in rapidity. The shaded bands show the scale uncertainties when using the recommended scale choice of $\mu =\hat{H}_\mathrm {T}$ for inclusive jets. The lower panel displays the ratio with respect to the NLO prediction

Closure tests for the inclusive jet $p_\mathrm {T}$ distribution are shown in Fig. 1 for two representative ranges in jet rapidity. It can be seen that the interpolation tables are able to reproduce the reference prediction at permille level, which is well below the experimental uncertainty and the residual Monte Carlo statistical uncertainty of the calculation itself. Slight systematic trends are observed, where the interpolation quality degrades somewhat towards more forward rapidity. This is to be expected as the forward region samples a wider range of the momentum fractions, $x_i$, thus introducing larger interpolation errors when the number of nodes is the same for all bins. In principle, the density of interpolation nodes can be adjusted in a phase-space dependent manner to mitigate this degradation, but this would result in larger file sizes and slower grid convolution. Closure tests were performed for all grids that are made available together with this publication, all of which show a similar level of interpolation quality that is typically below the permille level and reaches a maximum of 0.2% in exceptional phase-space regions.

### Scale, $\alpha _{\mathrm {s}}$, and PDF variations and their uncertainties

The predictions up to NNLO using the newly generated grids are shown in Fig. 2 for two representative rapidity ranges chosen for illustration. The bands correspond to the envelope from independently varying $\mu _\mathrm{R}$ and $\mu _\mathrm{F}$ up and down by factors of two with the constraint $\tfrac{1}{2}\le \mu _\mathrm{R}/\mu _\mathrm{F} \le 2$. A small resulting spread of the calculations is observed, with the successive orders displaying a satisfactory overlap within their respective uncertainty estimates and, importantly, a dramatic reduction in the width of the scale variation bands is observed to result from the inclusion of higher-order corrections.

**Fig. 3:**
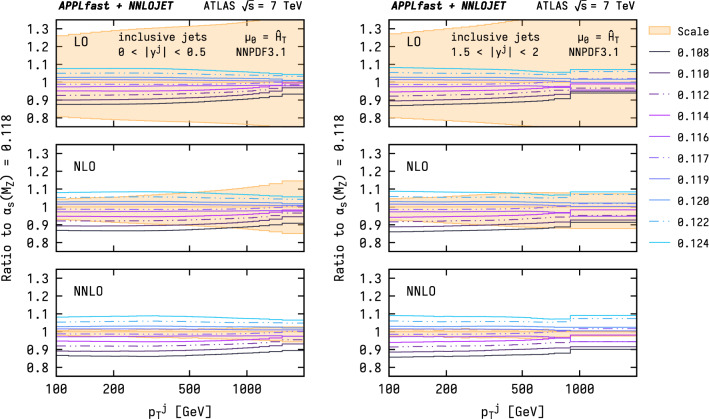
LO, NLO, and NNLO predictions for the ATLAS 7 TeV inclusive jet cross section as a function of $p^\mathrm {jet}_\mathrm {T}$ for two representative ranges in rapidity and for different values of $\alpha _{\mathrm {s}} (M_{{\mathrm {Z}}})$. The predictions are obtained with $\alpha _{\mathrm {s}}$-dependent variants of the NNPDF3.1 PDF set. The shaded area indicates the scale uncertainty at the given perturbative order

The sensitivity of the jet spectrum to the strong coupling, $\alpha _{\mathrm {s}}$, is presented in Fig. 3 for the different perturbative orders. To this end, the NNPDF3.1 PDF sets with different values for the strong coupling are used, covering the range between $0.108 \le \alpha _{\mathrm {s}} (M_{{\mathrm {Z}}}) \le 0.124$. It is observed that the scale variation at LO is substantially larger than that from varying $\alpha _{\mathrm {s}}$, while, at NLO, the spread in predictions from each of these two sources are similar. In contrast, the reduced scale-variation uncertainty at NNLO allows for the resolution of the variation of $\alpha _{\mathrm {s}}$ at the level of a few percent for the first time, illustrating the need for at least NNLO predictions for a robust extraction of $\alpha _{\mathrm {s}}$ at this level of precision.

**Fig. 4:**
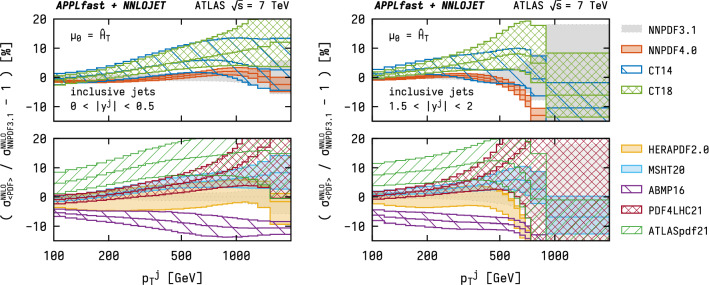
NNLO predictions for the ATLAS 7 TeV inclusive jet cross section as a function of $p^\mathrm {jet}_\mathrm {T}$ for two representative ranges in rapidity and for various different PDF sets. The shaded areas indicate the respective PDF uncertainties

The fast convolution that is made possible by using the grids allows the provision of NNLO predictions with different PDF sets, together with their complete respective uncertainties. Figure 4 contrasts the NNLO prediction using the NNPDF3.1 [36], NNPDF4.0 [38], CT14 [37], CT18 [39], HERAPDF2.0 [40], MSHT20 [41], ABMP16 [42], PDF4LHC21 [43], and ATLASpdf21 [44] PDF sets by showing the relative difference with respect to the prediction using the NNPDF3.1 set. With the exception of the ABMP16, HERAPDF2.0, and ATLASpdf21 sets, the predictions for the different PDF sets are mutually compatible within their respective uncertainty estimates. The PDF uncertainties are typically at the level of a few percent in the low-$p_\mathrm {T}$ regime and increase towards larger $p_\mathrm {T}$ (or rapidity *y*) to $\mathcal {O}(10\%)$ uncertainties in the $\mathrm {TeV}$ range.

### Robustness of NNLO $K$-factors

Owing to the large computational expenditure that NNLO calculations for jet production entail, their direct use in PDF fits has previously been unfeasible. Instead, a common approach proceeds by complementing predictions from NLO interpolation grids with an NNLO $K$-factor  [45]. The NNLO $K$-factor is a proxy for the full NNLO prediction and defined as

12 $$\begin{aligned} K^\text {NNLO} (\mu )&\equiv \frac{\mathrm {d}\sigma ^\text {NNLO} (\mu )/\mathrm {d}p_\mathrm {T}}{\mathrm {d}\sigma ^\text {NLO} (\mu )/\mathrm {d}p_\mathrm {T}} \, , \end{aligned}$$

where the dependence on $(\mu _\mathrm{R},\mu _\mathrm{F})$ is abbreviated with a single scale $\mu$ for simplicity. To this end, both the numerator and denominator are evaluated with the same PDF set. However, the NNLO $K$-factor can be applied in two different ways, namely

13a $$\begin{aligned} \sigma _\text {approx.\,1}^{\text {NNLO}} (\mu )&= \sigma ^{\text {NLO}} (\mu ) \times K^\text {NNLO} (\mu _\text {ref}) \quad \text {or} \end{aligned}$$

13b $$\begin{aligned} \sigma _\text {approx.\,2}^{\text {NNLO}} (\mu )&= \sigma ^{\text {NLO}} (\mu ) \times K^\text {NNLO} (\mu ) \, , \end{aligned}$$

the consequences of which will be discussed in the remainder of this section.

**Fig. 5:**
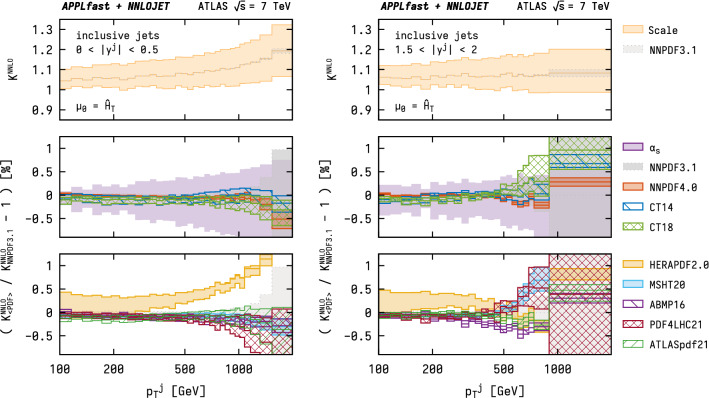
Summary of studies of the NNLO $K$-factor for the ATLAS 7 TeV inclusive jet cross section as a function of $p^\mathrm {jet}_\mathrm {T}$ for two representative ranges in rapidity. Top: the NNLO $K$-factor and corresponding scale dependence. Middle and bottom panels: $K$-factor for different PDF sets with their respective PDF uncertainty and dependence on the value of $\alpha _{\mathrm {s}} (M_{{\mathrm {Z}}})$, shown as a ratio with respect to that obtained with NNPDF3.1

The naïve application of a constant $K$-factor using the reference scale $\mu _\text {ref}$ as done in Eq. (13a) gives rise to a scale uncertainty that is determined by the NLO component and thus at the $\pm 5$–$10\%$ level for inclusive jets. As a consequence, fits and extractions of SM parameters that are based on this approach and incorporate scale variations as uncertainties will give rise to overly conservative estimates. In cases where these uncertainties are sizeable, such as $\alpha _{\mathrm {s}}$ extractions, a more reliable prediction is desirable.

The application in a scale-correlated manner as in Eq. (13b), on the other hand, allows for scale compensations to occur between $\sigma ^{\text {NLO}}$ and $K^\text {NNLO}$, and it is necessary that the $K$-factor be evaluated independently at both the central scale and all the other scales in question. This is, however, limited by the robustness of the $K$-factor with respect to changes of the PDFs, as the two terms in Eq. (13b) are in general evaluated using different PDF parameterisations. With the availability of NNLO grids, these assumptions can now be tested for any PDF sets, including their full uncertainties. Such a comparison is performed in Fig. 5 for two representative rapidity regions, where the top panel in each group shows $K^\text {NNLO} (\mu )$ as defined in Eq. (12). The corresponding scale dependence is added for illustrative purpose and shown as the yellow filled bands that exhibit relative variations of $\pm 5$–$10\%$. While this scale variation can be associated with the ambiguity in the choice of $\mu _\text {ref}$ in the naïve approach (13a), caution is advised in treating it as an additional uncertainty on top of the scale depende of $\sigma ^{\text {NLO}}(\mu )$ as it would lead to a substantial double-counting of the NLO-like scale variations. In the case of the scale-correlated approach (13b), it should be noted that the scale variations of $K^\text {NNLO} (\mu )$ displayed here will cancel to a large extent with the correlated variation in $\sigma ^{\text {NLO}}(\mu )$, and therefore will not subsist in the final prediction.

The lower two panels in Fig. 5 display the relative difference between different NNLO *K*-factors for different PDF sets with respect to the baseline NNPDF3.1 prediction. The middle panel further includes a shaded band (purple) that indicates the sensitivity to $\alpha _{\mathrm {s}}$, again using variations in the range $0.108 \le \alpha _{\mathrm {s}} (M_{{\mathrm {Z}}}) \le 0.124$. Overall, a remarkable robustness of the NNLO $K$-factor with respect to changes in the PDF set is observed at lower $p_\mathrm {T}$, agreeing to within the 0.5% level. The largest excursions are again seen for the ABMP16 and HERAPDF2.0 sets that, nevertheless, still remain typically within $\pm 0.5$–$1\%$. The PDF uncertainties within a given PDF set are at a similar level. These results indicate that the approach following Eq. (13b) is, in general, likely to be safe also for fitting applications where the $K$-factors have been calculated independently for all of the required scales. Nonetheless, the availability of interpolation grids frees us from having to rely on such an assumption.

### The total fiducial inclusive jet cross section

The total fiducial inclusive jet cross section, $\sigma _\text {tot}^\text {jet}$, is one of the largest inelastic cross sections measured in proton–proton collisions and, as such, it is an important process for QCD studies. Moreover, this process forms an important QCD induced background for many other processes measured at the LHC, so it is important to have a precise knowledge of the size of this cross section, and of the theoretical uncertainties, in order to maximise the precision and physics potential of measurements from hadron–hadron colliders.

For this study, the total jet cross section is defined as the single-jet inclusive cross section within a selected rapidity interval $|y^\text {jet}|$ and for a minimal transverse momentum of $p^\mathrm {jet}_\mathrm {T,min}$. The ATLAS Collaboration has measured total jet cross sections for anti-$k_\text {T}$ jets with $R=0.4$ in the range $p^\mathrm {jet}_\mathrm {T} >100\,$GeV and $|y^\text {jet}|<3.0$ at centre-of-mass energies of 7, 8, and 13 TeV [28, 32, 34, 46].^2^ For the CMS Collaboration, the total jet cross section for centre-of-mass energies of 2.76, 7, 8, and 13 TeV is derived from double-differential measurements for anti-$k_\text {T}$ jets with $R=0.7$ [30, 33, 35, 48] by summing the cross sections in the bins of the common fiducial phase space of $p^\mathrm {jet}_\mathrm {T} >97$ GeV and $|y^\text {jet}|<2.0$. The experimental uncertainties are obtained by propagating each uncertainty component individually and accounting for correlations. The displayed total experimental uncertainty is then obtained by quadratic addition of all uncertainty components.

**Fig. 6:**
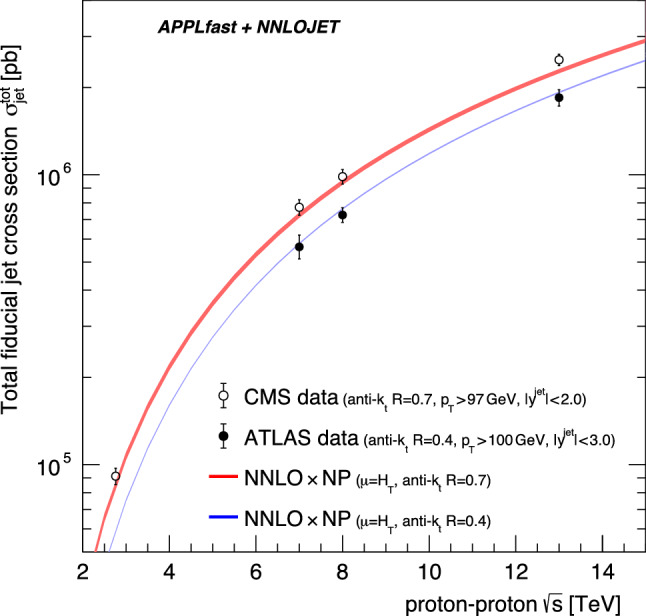
The total jet cross section as a function of the proton–proton centre-of-mass energy for anti-$k_\text {T}$ jets with $R=0.4$ and 0.7. The predictions are compared to data from ATLAS (for $R=0.4$) and CMS (for $R=0.7$), and the fiducial region is selected according to the available data. The size of the shaded area indicates the scale uncertainty, evaluated as described in the text

Applying the technique of centre-of-mass reweighting [8] for a grid, only the single grid at the largest centre-of-mass energy of $\sqrt{s}=13$ TeV is required for each jet-*R* cone size. For the NNLO predictions the PDF4LHC21 PDF set is used with the recommended scale of $\mu _\mathrm{R/F} =\hat{H}_\mathrm {T}$. Non-perturbative correction factors are taken from the relevant experimental publications as cross-section weighted averages and are applied to the NNLO predictions. Lacking bin-to-bin correlations, uncertainties for the non-perturbative corrections have not been derived. Figure 6 presents the results for the total jet cross section in comparison to data as a function of the centre-of-mass energy for $R=0.4$ and $R=0.7$. A reasonable agreement is observed between the data and the predictions for all centre-of-mass energies. The $\sqrt{s}$-dependence of the data is also well reproduced by the NNLO predictions.

**Fig. 7:**
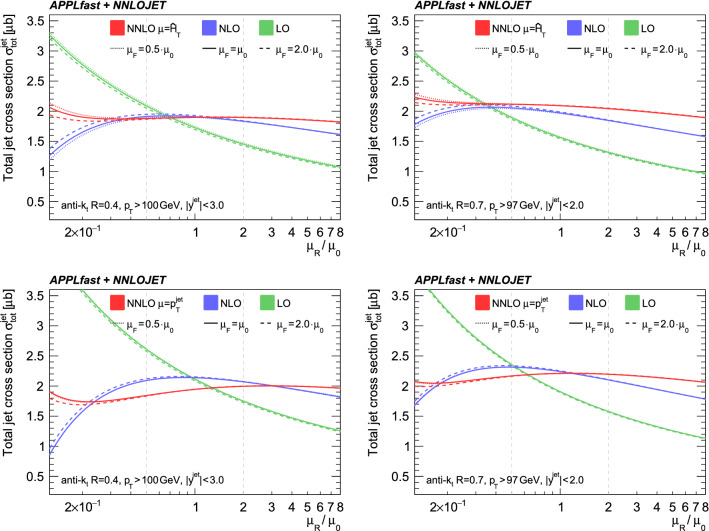
The scale dependence of the total jet cross section at $\sqrt{s}=13$ TeV for $R=0.4$ (left column) and $R=0.7$ (right column) anti-$k_\text {T}$ jets. The top row presents the scale dependence with $\mu _\mathrm{R/F} = \hat{H}_\mathrm {T}$ as central scale, while the bottom row is for $\mu _\mathrm{R/F} =p^\mathrm {jet}_\mathrm {T}$

In the top row of Fig. 7 the scale dependence of $\sigma _\text {tot}^\text {jet}$ for $R=0.4$ and 0.7 at $\sqrt{s}=13$ TeV is presented for $\mu _\mathrm{R/F} =\hat{H}_\mathrm {T}$. Shown is the total jet cross section at LO, NLO, and NNLO, where scale factors ranging from 0.125 to 8 are applied to $\mu _\mathrm{R}$, shown on the horizontal axis, and for the three scale factors of 0.5, 1, and 2 for $\mu _\mathrm{F}$ shown in the band. The NNLO correction is smaller than the NLO correction, and, as expected, the scale dependence decreases moving from LO to NLO to NNLO. Both the NLO and the NNLO correction are somewhat smaller for the cone size $R=0.4$ as compared to $R=0.7$. For comparison, the scale dependence is also shown when using $\mu =p^\mathrm {jet}_\mathrm {T}$ in the bottom row of Fig. 7. In this case the NLO and the NNLO corrections decrease, but remain larger than unity for the cone size of $R=0.7$, while they become very small at NLO and even smaller than unity at NNLO for $R=0.4$. Moreover, the scale uncertainty at NNLO becomes larger than the one at NLO for the smaller cone size with $\mu =p^\mathrm {jet}_\mathrm {T}$ confirming the findings of Ref. [23].

Even though the predictions for the total jet cross section exhibit smaller scale uncertainties and smaller NNLO $K$-factors for $R=0.4$ than for $R=0.7$ for the recommended scale $\mu =\hat{H}_\mathrm {T}$, non-perturbative corrections have to be considered as well for jet transverse momenta as small as $100\,$GeV. For the cone radius $R=0.4$ the non-perturbative correction is close to unity, while for $R=0.7$ it is of the order of 8% [34, 35]. The uncertainties on these corrections, however, are larger for the small cone size.

**Fig. 8:**
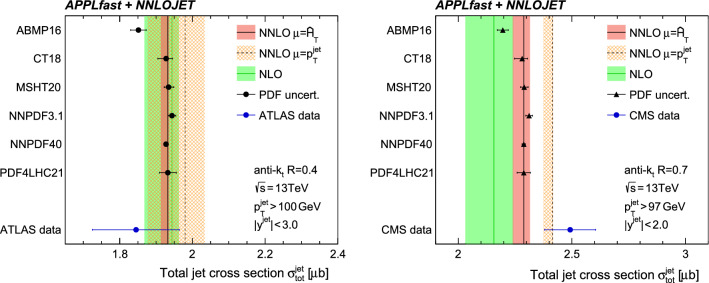
The PDF dependence of the total jet cross sections for $R=0.4$ (left) and 0.7 (right) for the fiducial regions defined for ATLAS and CMS, respectively. Uncertainties of a few percent from non-perturbative corrections are not available for the total jet cross sections

The total jet cross section is also an important benchmark process for PDF determinations, since it is sensitive to the gluon content of the proton. In Fig. 8 the predicted total cross section $\sigma _\text {tot}^\text {jet}$ for various PDF sets (error bars indicating the respective PDF uncertainties only) is presented. Without accounting for other theoretical uncertainties—in particular those from non-perturbative corrections—it can be observed that at $R=0.4$ the predictions are in agreement with the data, while for the larger $R=0.7$ radius the resulting cross section found using any of the PDF sets underestimates the measurement from CMS.

**Table 2:** An overview of dijet datasets with APPLfast interpolation grids for proton–proton collisions at the LHC. For each dataset the centre-of-mass energy $\sqrt{s}$, the integrated luminosity $\mathcal {L}$, the number of data points, and the jet algorithm are listed. The two leading jets must fulfil the requirements with respect to their rapidities $y_1, y_2$ and transverse momenta $p_{\mathrm {T},1}$, $p_{\mathrm {T},2}$. In addition, the choice of scale for $\mu _\mathrm{R/F}$ in the interpolation grids is shown

Data	$\sqrt{s}$ [TeV]	$\mathcal {L} [\mathrm{fb}^{-1}]$	No. of points	Anti-$k_\text {T}$ *R*	Kinematic range [GeV]	Fiducial cuts	$\mu _\mathrm{R/F}$-choice
ATLAS [55]	7.0	4.5	90	0.6	$m_\text {12} \in [260,5040]$	$|y_1|,|y_2|<3.0$	$m_\text {12}$
						$$[p_{\mathrm {T},1},p_{\mathrm {T},2} ]>[100,50]\mathrm {GeV} $$	
						$$y^{*} <3.0$$	
CMS [31]	7.0	5.0	54	0.7	$m_\text {12} \in [197,5058]$	$\left| y\right| <5.0$	$m_\text {12}$
						$$[p_{\mathrm {T},1},p_{\mathrm {T},2} ]>[60,30]\mathrm {GeV} $$	
						$$|y_{\text {max}} |<2.5$$	
CMS [49]	8.0	19.7	122	0.7	$\langle p_\text {T1,2}\rangle \in [133,1784]$	$\left| y\right| <5.0$	$p_{\mathrm {T},1} \exp (0.3\,y^{*})$
						$$p_{\mathrm {T},1},p_{\mathrm {T},2} >50\mathrm {GeV} $$	
						$|y_1|,|y_2|<3.0$	$m_\text {12}$
ATLAS [34]	13.0	3.2	136	0.4	$m_\text {12} \in [260,9066]$	$|y_1|,|y_2|<3.0$	$m_\text {12}$
						$$p_{\mathrm {T},1},p_{\mathrm {T},2} >75\mathrm {GeV} $$	
						$$\langle p_\text {T1,2}\rangle >100\mathrm {GeV} $$	
						$$y^{*} <3.0$$	

The predictions using the various PDF sets are mutually compatible with the exception of the ABMP16 PDF set, which predicts a significantly smaller cross section. The PDF uncertainties exhibited by the different PDF sets vary in size by approximately a factor of two. The recent NNPDF4.0 PDF set, however, estimates PDF uncertainties to be significantly smaller than the others.

Also seen in Fig. 8 are the predictions at NLO and NNLO with scale uncertainty bands. As expected from the discussion of Fig. 7 the NNLO $K$-factor is close to unity for an anti-$k_\text {T}$ jet radius of $R=0.4$, and larger for $R=0.7$. Within the scale uncertainties, however, the predictions at NLO and NNLO remain compatible with each other. In contrast, for $R=0.7$ the NNLO predictions for the alternative scale of $\mu _\mathrm{R/F} =p^\mathrm {jet}_\mathrm {T}$ do not lie within the NLO scale uncertainty.

## PDF and $\alpha _{\mathrm {s}}$ fits using dijet data

In this section some of the full capabilities of the interpolation grids are illustrated by performing a PDF fit using a single dijet dataset from the measurements listed in Table 2; specifically, the CMS triple-differential dijet data at $\sqrt{s}=8\,\mathrm {TeV}$ [49]. Since this measurement has already been used in simultaneous PDF and $\alpha _{\mathrm {s}} (M_{{\mathrm {Z}}})$ fits at NLO QCD, an example of those results [50], is first reproduced, and then subsequently augmented by the inclusion of NNLO predictions. The three dimensions of this dataset divide the phase space into bins of the average transverse momentum of the two leading $p_\mathrm {T}$ jets, $\langle p_\text {T1,2}\rangle = (p_{\mathrm {T},1} +p_{\mathrm {T},2})/2$, the longitudinal boost of the dijet system given by half the sum of the leading jet rapidities, $y_{\mathrm {b}} = |y_1+y_2|/2$, and half their rapidity separation, $y^{*} = |y_1-y_2|/2$, which is related to the scattering angle of the jets in the centre-of-mass system.

It should be noted that the omission of sub-leading colour effects in the NNLO part of the calculation, as utilised throughout this work, could potentially have a more sizeable effect in the dijet process. In particular, in the calculation of the triple-differential cross section an effect of up to $\pm 5\%$ was observed in the $\langle p_\text {T1,2}\rangle$ spectrum [26].

All PDF fits presented here are performed using xFitter^3^ [51–53] version 2.0.1, with technical updates required to fully exploit the new NNLO grids, as described in Ref. [54]. The details of the fits closely follow the HERAPDF2.0 methodology [40], with adaptions as described in Ref. [50]. Further specific details of the fit parameterisation and procedure are described in the following subsections.

### Reproduction of previous fits at NLO

In order to validate the PDF fitting procedure used for this analysis, two NLO fits are first performed: one using the HERA I+II inclusive DIS data alone, and another that additionally includes the CMS triple-differential dijet data.

As prescribed by the HERAPDF2.0 procedure, the PDFs $f_i(x)$ are parameterised at some starting scale $\mu _{\mathrm{F}_0}$ by

14 $$\begin{aligned} xf_i(x) = A_i x^{B_i} (1-x)^{C_i} (1+{D_i}x+{E_i}x^2)\,, \end{aligned}$$

where the parameters $A_i$, $B_i$, and $C_i$ are always included, while the $D_i$ and $E_i$ parameters increase the flexibility of the fit and can be used to estimate the parameterisation uncertainties. To describe the proton, five such PDFs are parameterised, defined here to be: the gluon $f_\text {g}$; the valence quarks $f_{\text {u}_\text {v}}=f_\text {u}-f_{\overline{\text {u}}}$ and $f_{\text {d}_\text {v}}=f_\text {d}-f_{\overline{\text {d}}}$; and the light up- and down-type anti-quark distributions $f_{\overline{\text {U}}}=f_{\overline{\text {u}}}$ and $f_{\overline{\text {D}}}=f_{\overline{\text {d}}}+f_{\overline{\text {s}}}$. It should be noted that the default HERAPDF2.0 parameterisation, also used in Ref. [49], includes a second subtracted term of the form $A'_\text {g} x^{B'_\text {g}} \left( 1-x\right) ^{C'_\text {g}}$ for the gluon distribution, for fits beyond LO. Following Ref. [50], this term is not adopted here, since it offers no advantage in $\chi ^2/n_\text {dof}$ for the performed studies.

Of the five normalisation constants $A_i$, three are constrained by the quark-number and momentum sum rules. Following HERAPDF2.0 choices, a symmetric low-*x* behaviour of the up-and down-type quark sea is assumed, the strange sea distribution is written as a fixed fraction $f_{\overline{\text {s}}/\overline{\text {D}}}=0.4$ of the down-type quark sea,^4^ and it is assumed that $x\text {s}=x\overline{\text {s}}$. Finally, the $\overline{\text {u}}$ and $\overline{\text {d}}$ anti-quark normalisations are constrained to be equal in the limit $x\rightarrow 0$. This would leave ten free parameters if all $D_i$ and $E_i$ were set to zero.

Following Ref. [50], specific differences with respect to the published HERAPDF2.0^5^ are: a larger minimum $Q^2$ cut for the DIS data of $Q^2_\text {min}=7.5\,\mathrm {GeV} ^2$, the non-inclusion of the negative gluon term, and a choice of a 13-parameter fit at NLO, with the parameters $E_\text {g}$, $D_{\text {u}_\text {v}}$ and $D_{\overline{\text {U}}}$ included, as these were found to optimally fit the CMS triple-differential dijet data when added to the minimal set of ten parameters. Additional differences with respect to the PDF fit described in the CMS publication [49] are summarised in Appendix A. The theoretical calculation used to fit the CMS dijet measurement is from NLOJet++  [56, 57], encoded in the fast interpolation grids of fastNLO.

The starting values of the parameters for the fit to HERA DIS data alone are set to those published by the HERAPDF2.0 analysis [40], except for the parameters $E_\text {g}$ and $D_{\text {u}_\text {v}}$, which were not fitted there and are given a starting value of 0. The values of all 13 parameters resulting from this fit are then used as starting values for the simultaneous fit to the HERA DIS and the CMS dijet data.

Following Ref. [50]^6^, the CMS dijet data used were limited to the range $\langle p_\text {T1,2}\rangle < 1\,\mathrm {TeV}$ and without electroweak corrections. The results are in good agreement with those in Ref. [50]. Since electroweak corrections are now available, the NNLO fit presented in Sect. 4.2 includes these, and uses the full $\langle p_\text {T1,2}\rangle$ range of the measurement. For validation, an additional fit at NLO was performed including the CMS $\langle p_\text {T1,2}\rangle$ data beyond $1\,$TeV, with electroweak corrections applied, and taking into account two additional uncertainty sources, as described in Appendix A. It was found that these modifications lead to only negligible differences, supporting the contention that their impact is limited, due to the larger statistical uncertainties on the jet data at high scales, and the small size of the electroweak corrections at smaller scales. Replacing the NLO prediction from NLOJet++ with that from  leaves the results practically unchanged, as expected. Further details can be found in Ref. [54].

### Extension to NNLO

Following the initial validation of the fit procedure to reproduce the previous fit, the methodology was extended to include the NNLO predictions available from . The corresponding interpolation grids have been created with two different central scale choices: $\mu _\mathrm{R/F} =p_{\mathrm {T},1} \exp (0.3\,y^{*})$ as before, and $\mu _\mathrm{R/F} =m_\text {12}$, the mass of the dijet system, as recommended in Ref. [25]. To be less sensitive to potential issues of the theoretical description at low *x*, such as the need for resummation corrections [58] or the impact of higher-twist corrections at low $Q^2$, the minimum $Q^2$ for the DIS data is increased to $Q^2_\text {min}=10.0\,\mathrm {GeV} ^2$.

**Fig. 9:**
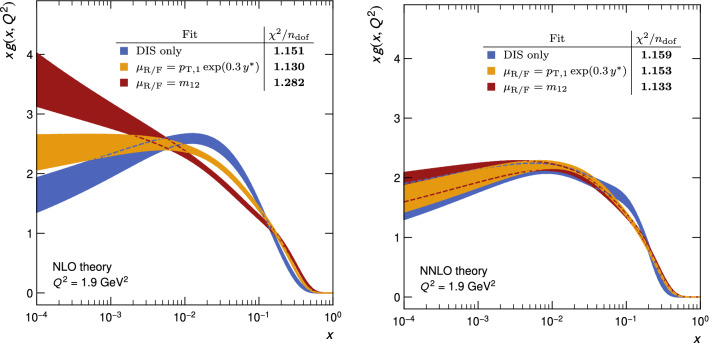
The gluon PDF from fits to HERA DIS alone and with CMS dijet data at NLO (left) and NNLO (right) for two different central scale choices for dijet production: $\mu _\mathrm{R/F} =p_{\mathrm {T},1} \exp (0.3\,y^{*})$ (yellow) and $\mu _\mathrm{R/F} =m_\text {12}$ (red). Only the experimental uncertainties are shown

By examining the gluon distribution – the most sensitive parton distribution – the dependence of the fit result on the central scale choice is investigated, as shown in Fig. 9. At NLO (left), significant differences are observed for the two scales, while at NNLO (right) all fit qualities improve and the differences resulting from the two scales are drastically reduced such that the uncertainty bands overlap even though they represent only the experimental uncertainty. It is perhaps interesting to note that the recommended scale $\mu _\mathrm{R/F} =m_\text {12}$ exhibits, at NLO, a significantly worse $\chi ^2/n_\text {dof}$ of 1.282 than the value 1.130 obtained using the scale $p_{\mathrm {T},1} \exp (0.3\,y^{*})$, while at NNLO the scale choice of $m_\text {12}$ exhibits the best value of $\chi ^2/n_\text {dof}$, close to unity.

**Fig. 10:**
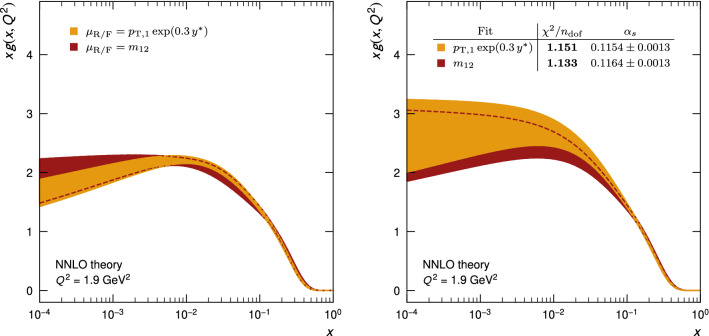
Fits at NNLO of the gluon PDF using HERA DIS and CMS dijet data, but with uncertainties including scale variations (left) as described in the text, or with $\alpha _{\mathrm {s}} (M_{{\mathrm {Z}}})$ as an additional parameter (right)

Employing the conventional scale variation methodology as a proxy for the effect of missing higher orders, the fits for both scales overlap as demonstrated in Fig. 10 (left), where the scale variations are considered as an additional uncertainty in the form of an envelope (evaluated using the “offset method”), added quadratically to the experimental uncertainty. Detailed fit results can be found in Table 5 in Appendix A, and in Ref. [54], where also a simultaneous fit to both CMS dijet datasets of Table 2 is discussed.

**Table 3:** Values of $\alpha _{\mathrm {s}} (M_{{\mathrm {Z}}})$ determined in the (13+1)-parameter PDF+$\alpha _{\mathrm {s}} (M_{{\mathrm {Z}}})$ fit at NLO and at NNLO with HERA DIS and CMS triple-differential dijet data, as described in the text

Order	Scale choice	$\alpha _{\mathrm {s}} (M_{{\mathrm {Z}}})$	$\varDelta \alpha _{\mathrm {s}} (M_{{\mathrm {Z}}}) \cdot 10^4$				
			(Exp)	(Scale)	(Model)	(Param)	(Total)
NLO	$p_{\mathrm {T},1} \exp (0.3\,y^{*})$	0.1192	$\pm 15$	$^{+26}_{-9}$	$^{+5}_{-4}$	$^{+1}_{-3}$	$^{+31}_{-19}$
	$m_\text {12}$	0.1210	$\pm 15$	$^{+32}_{-27}$	$\pm 4$	$^{+6}_{-7}$	$^{+36}_{-32}$
NNLO	$p_{\mathrm {T},1} \exp (0.3\,y^{*})$	0.1154	$\pm 13$	$^{+7}_{-8}$	$^{+5}_{-4}$	$^{+1}_{-7}$	$^{+15}_{-17}$
	$m_\text {12}$	0.1164	$\pm 13$	$^{+11}_{-5}$	$^{+6}_{-4}$	$^{+2}_{-6}$	$^{+18}_{-16}$

### Full fit of PDFs and $\alpha _{\mathrm {s}} (M_{{\mathrm {Z}}})$

The procedure established in Sect. 4.2 is extended further by including $\alpha _{\mathrm {s}} (M_{{\mathrm {Z}}})$ as an additional free parameter in the fit. This leads to similar results for the PDFs as before, but with larger uncertainties, in particular for the gluon, shown in Fig. 10 (right).

Table 3 shows the values of $\alpha _{\mathrm {s}} (M_{{\mathrm {Z}}})$ obtained for the simultaneous PDF and $\alpha _{\mathrm {s}} (M_{{\mathrm {Z}}})$ fits. It is observed that the values preferred by the NNLO fits are smaller than those obtained at NLO. The fit quality, compared in the inserts in Figs. 9 (right) and 10 (right), is largely unchanged, since the previously fixed value of $\alpha _{\mathrm {s}} (M_{{\mathrm {Z}}}) =0.1180$ is already close to the minimum found here. The experimental uncertainty on the quoted value of $\alpha _{\mathrm {s}} (M_{{\mathrm {Z}}})$ is determined from the parabolic dependence of the $\chi ^2$ function near the minimum, and the scale uncertainty is obtained as described in Sect. 4.2, using the offset method.

Two additional sets of uncertainties are estimated, to account for the chosen PDF parameterisation and the values of certain model and procedural choices, the latter detailed in Table 6 of Appendix A. The parameterisation uncertainty is determined by including all the remaining *D* and *E* parameters in the fit one at a time and taking the largest positive and negative difference of any of these variations, with respect to the nominal $\alpha _{\mathrm {s}} (M_{{\mathrm {Z}}})$ value, as an asymmetric uncertainty. To obtain the model uncertainty, fits are performed for up and down variations of the masses of the charm quark, $m_\text {c}$, and bottom quark, $m_\text {b}$, the strangeness fraction, $f_{\overline{\text {s}}/\overline{\text {D}}}$, the starting scale, $\mu _{\text {F}_0}$, and the $Q^2_\text {min}$ cut imposed on the DIS data. Table 6 lists the values of the parameters used in the nominal fit and for the systematic variations. The signed differences with respect to the nominal $\alpha _{\mathrm {s}} (M_{{\mathrm {Z}}})$ value, resulting from the variation of each parameter, are added in quadrature to yield the overall model uncertainty. The final result using the recommended scale choice of $\mu _\mathrm{R/F} =m_\text {12}$ is $\alpha _{\mathrm {s}} (M_{{\mathrm {Z}}}) = 0.1164\,^{+0.0018}_{-0.0016}\,\mathrm {(tot)}$, which is compatible with the result using the alternative scale choice (see Table 3) and the world average of $\alpha _{\mathrm {s}} (M_{{\mathrm {Z}}}) = 0.1179 \pm 0.0009$ [59].

## Conclusions and outlook

The technique of interpolation grids has been proven to be an indispensable tool for QCD phenomenology of hadron-collider data, since it allows repeated calculations of pQCD cross sections with varying input conditions such as scale choices, parton distribution functions, or the strong coupling, $\alpha _{\mathrm {s}}$. The grid technique is implemented in the APPLgrid and fastNLO computer codes and, for this paper, a common interface for both packages with the  computer program for hadron–hadron processes has been developed, enabling the generation of fast interpolation grids at next-to-next-to-leading order in QCD for inclusive jet and dijet production cross sections at the LHC.

The performance of the grid technique for selected LHC jet datasets is presented, demonstrating a closure of those interpolation grids generated for inclusive jet and dijet cross sections to generally better than 0.1%. Although the NNLO calculations for jet production are computationally very expensive, all grids were generated such that the statistical uncertainty is less than 1% in most cases, but may increase to 2–4% for some regions near the edges of the phase space from the measurement.

The grids have been employed for phenomenological studies that are otherwise computationally prohibitive. The NNLO predictions for inclusive jet and dijet cross sections were evaluated for different PDF sets including the full PDF uncertainties. It is found that the PDFs from the CT, MSHT, and NNPDF global fitting groups yield largely consistent predictions for jet production cross sections over a large kinematic range in $p^\mathrm {jet}_\mathrm {T}$ and jet rapidity *y*. The predictions using the ABMP16 or HERAPDF2.0 PDFs exhibit some deviations beyond the PDF uncertainty bands, e.g. at large $p^\mathrm {jet}_\mathrm {T}$ or *y*. The new NNPDF4.0 set yields surprisingly small PDF uncertainties compared to the other available PDF sets.

Predictions for NNLO cross sections are often included in QCD phenomenological studies through the use of NNLO $K$-factors. Here, their stability while varying the strong coupling $\alpha _{\mathrm {s}}$ or the PDF set has been studied. Overall, it is observed that the NNLO $K$-factors are largely insensitive to the choice of $\alpha _{\mathrm {s}}$ as expected, but exhibit a dependence on the PDF sets at the few permille level in the bulk of phase space that increases to the percent level towards the tails of the $p_\mathrm {T}$ distributions. The latter are sub-dominant compared to NNLO scale uncertainties, though not necessarily negligible. Therefore, although a case could be made to not require the full grid for reproduction of the central cross section, for full precision and, in particular, for the evaluation of the scale uncertainties, the use of interpolation grids is preferred over a $K$-factor approach.

**Table 4:** Partial $\chi ^2$ values for the NLO fits of Sect. 4.2

HERA I+II	Combined	$n_\mathrm {data}$	HERA I+II	With CMS dijets	With CMS dijets
			DIS only	$\mu _\mathrm{R/F} =p_{\mathrm {T},1} \exp (0.3\,y^{*})$	$\mu _\mathrm{R/F} =m_\text {12}$
		1016	1106.14	1124.45	1157.94
CMS $8\,\mathrm {TeV}$ dijets	yb0 ys0	31	–	14.58	29.13
	yb0 ys1	26	–	11.36	22.41
	yb0 ys2	14	–	17.77	52.60
	yb1 ys0	23	–	11.29	20.41
	yb1 ys1	17	–	18.87	21.69
	yb2 ys0	11	–	19.51	58.30
	combined	122	–	93.38	204.54
Correlated $\chi ^2$			50.96	65.04	92.11
Log penalty $\chi ^2$			$-$ 2.98	$-$ 11.39	$-$ 12.89
Combined			1154.12	1271.48	1441.72
$n_\text {dof}$			1003	1125	1125
p value			$6.13\times 10^{-4}$	$1.45\times 10^{-3}$	$3.86\times 10^{-10}$
Combined $\chi ^2/n_\text {dof}$			1.151	1.130	1.282

**Table 5:** Partial $\chi ^2$ values for the NNLO fits of Sect. 4.2

HERA I+II	Combined	$n_\mathrm {data}$	HERA I+II	With CMS dijets	With CMS dijets
			DIS only	$\mu _\mathrm{R/F} =p_{\mathrm {T},1} \exp (0.3\,y^{*})$	$\mu _\mathrm{R/F} =m_\text {12}$
		1016	1109.15	1115.65	1124.45
CMS $8\,\mathrm {TeV}$ dijets	yb0 ys0	31	–	18.15	14.58
	yb0 ys1	26	–	13.07	11.36
	yb0 ys2	14	–	26.63	17.77
	yb1 ys0	23	–	14.45	11.29
	yb1 ys1	17	–	23.91	18.87
	yb2 ys0	11	–	18.19	19.51
	Combined	122	–	114.40	93.38
Correlated $\chi ^2$			55.48	65.78	61.47
Log penalty $\chi ^2$			$-$ 1.74	1.45	$-$ 1.67
Combined			1162.89	1297.30	1274.63
$n_\text {dof}$			1003	1125	1125
p value			$3.24\times 10^{-4}$	$2.55\times 10^{-4}$	$1.19\times 10^{-3}$
Combined $\chi ^2/n_\text {dof}$			1.159	1.153	1.133

The extension of the grid framework to hadron–hadron collider processes shown here further enables the generation of interpolation grids for the large set of processes available within . Such applications and phenomenological studies will be left for future studies.

The grids used in this analysis correspond to a large number of the available jet measurements from the ATLAS and CMS collaborations. They have been made available for the wider community on the Ploughshare  [19] website.

## Data Availability

The manuscript has associated data in a data repository. [Authors’ comment: The data generated in the context of this publication is comprised of the fast interpolation grids at NNLO accuracy. They are publicly available on the designated platform at ploughshare.web.cern.ch and can be freely downloaded and used to reproduce all results from the manuscript.]

## Appendix A: Details on the dijet fits

Additional setup differences in the reproduction fits of Sect. 4.1 with respect to the PDF fit described in the CMS publication Ref. [49] are:

1. A newer version of the fitting framework, xFitter v2.0.1, is used in preference to HERAFitter v1.1.1.
2. The parameter fixing the strange sea fraction, $f_{\overline{\text {s}}/\overline{\text {D}}}$, is set to 0.4 instead of 0.31.
3. At the time of writing of the PhD thesis Ref. [50] electroweak correction factors were not available. The range in $\langle p_\text {T1,2}\rangle$ for fits hence was restricted to $\langle p_\text {T1,2}\rangle < 1\,\mathrm {TeV}$.
4. The data comprise an additional experimental source of uncertainty that accounts for non-Gaussian tails in the jet energy resolution.
5. The statistical and numerical precision of the theory calculations is taken into account as an additional bin-to-bin uncorrelated uncertainty in the fit.
6. New dijet predictions at NLO pQCD have been calculated with interpolation grids produced with  [24] instead of NLOJet++  [56, 57].

The detailed results of the dijet fits of Sect. 4.2 are presented here in Tables 4 and 5. The labels “yb0 ys0” up to “yb2 ys0” denote the six measurement bins in $y_{\mathrm {b}}$ and $y^{*}$ with 0, 1, 2 corresponding to $0 < y_{\mathrm {b}} |y^{*}$, $1 \le y_{\mathrm {b}} |y^{*} < 2$, and $2 \le y_{\mathrm {b}} |y^{*} < 3$.

The variations used for the model uncertainties are summarised in Table 6.

**Table 6:** Values of the model parameters used in the fits in Sects. 4.2 and 4.3, and the corresponding systematic variations used to determine the model uncertainty on $\alpha _{\mathrm {s}} (M_{{\mathrm {Z}}})$ in Sect. 4.3. Omitted values are due to the restriction $\mu _{\text {F}_0} < m_\text {c}$ imposed by the RTOPT heavy flavour number scheme used here [60–62]. In these cases, to calculate the corresponding contributions to the model uncertainty, the symmetrised variation in the opposite direction is taken

Parameter	Central value	Variation	
		Down	Up
$f_{\overline{\text {s}}/\overline{\text {D}}}$	0.4	0.3	0.5
$m_\text {c}$ (GeV)	1.42	–	1.49
$m_\text {b}$ (GeV)	4.5	4.25	4.75
$\mu _{\text {F}_0}^2$ (GeV$^2$)	1.9	1.6	–
$Q^2_\text {min}$ (GeV$^2$)	10	7.5	12.5

## Footnotes

1 Note that all predictions provided in this manuscript are based on the leading-colour and leading $N_f$ approximation and do not include the most recent sub-leading colour contributions presented in Ref. [26].

2 The ATLAS Collaboration has also measured inclusive jet differential cross sections at $\sqrt{s}=2.76$ TeV [47], but the bin boundaries chosen for that measurement preclude it from being used here.

3 Formerly known as HERAFitter.

4 Reference [40] labels this parameter simply as $f_s$, which differs from the PDF notation adopted here.

5 The default HERAPDF2.0, as published, uses $Q^2_\text {min}=3.5\,\mathrm {GeV} ^2$, and a 14-parameter fit: 10 $+\, D_{\overline{\text {U}}}$, $E_{\text {u}_\text {v}}$, $A'_{\text {g}}$ and $B'_{\text {g}}$.

6 At the time of Ref. [50], electroweak corrections were not available, and so the CMS dijet data were restricted to a phase space region where these were expected to be small – see Appendix A.
